# Effects of Zeaxanthin on Growth and Invasion of Human Uveal Melanoma in Nude Mouse Model

**DOI:** 10.1155/2015/392305

**Published:** 2015-11-22

**Authors:** Xiaoliang L. Xu, Dan-Ning Hu, Codrin Iacob, Adrienne Jordan, Sandipkumar Gandhi, Dennis L. Gierhart, Richard Rosen

**Affiliations:** ^1^Department of Pathology, Memorial Sloan-Kettering Cancer Center, 1275 York Avenue, New York, NY 10021, USA; ^2^Department of Ophthalmology, The New York Eye and Ear Infirmary of Mount Sinai, Icahn School of Medicine at Mount Sinai, New York, NY 10003, USA; ^3^Department of Pathology, The New York Eye and Ear Infirmary of Mount Sinai, Icahn School of Medicine at Mount Sinai, New York, NY 10003, USA; ^4^ZeaVision LLC, Chesterfield, MO 63005, USA

## Abstract

Uveal melanoma cells were inoculated into the choroid of nude mice and treated with or without intraocular injection of zeaxanthin. After 21 days, mice were sacrificed and the eyes enucleated. Histopathological analysis was performed in hematoxylin and eosin stained frozen sections. Melanoma developed rapidly in the control group (without treatment of zeaxanthin). Tumor-bearing eye mass and tumor mass in the control group were significantly greater than those in zeaxanthin treated group. Melanoma in the controlled eyes occupied a large part of the eye, was epithelioid in morphology, and was with numerous mitotic figures. Scleral perforation and extraocular extension were observed in half of the eyes. Melanomas in zeaxanthin treated eyes were significantly smaller with many necrosis and apoptosis areas and no extraocular extension could be found. Quantitative image analysis revealed that the tumor size was reduced by 56% in eyes treated with low dosages of zeaxanthin and 92% in eyes treatment with high dosages of zeaxanthin, as compared to the controls. This study demonstrated that zeaxanthin significantly inhibits the growth and invasion of human uveal melanoma in nude mice, suggesting that zeaxanthin may be a promising agent to be explored for the prevention and treatment of uveal melanoma.

## 1. Introduction

Uveal melanoma is the most common malignant intraocular tumor in adults. It accounts for 80% of all noncutaneous melanomas. Up to 50% of uveal melanoma patients die from metastatic disease within 10 years of initial diagnosis and it accounts for 13% of all deaths caused by melanoma [1, 2]. Chemotherapy has had little or no success in both primary and metastatic uveal melanoma [3]. Therefore, it is an urgent necessity to develop more efficient and novel therapeutic agents for improving the survival of uveal melanoma patients.

Zeaxanthin is a nontoxic xanthophyll present in fruits and leafy green vegetables. Zeaxanthin is an antioxidant and can absorb blue light like a yellow filter. It has been used as a nutrition supplement for patients with various ocular diseases [4–9]. In addition to these effects, zeaxanthin may influence the viability and function of cells through various signal pathways or transcription factors [7]. It has been reported that higher intake and higher blood levels of zeaxanthin appear to be associated with a lower risk of occurrence of various cancers [10].

Our previous study demonstrated that zeaxanthin inhibits the proliferation and induces apoptosis of human uveal melanoma cells through intrinsic apoptosis pathway [10]. To our best knowledge, the effects of zeaxanthin on uveal melanoma in experimental animal models have not been reported previously. In the present study, we examined the effects of zeaxanthin on the growth and invasion of human uveal melanoma in an immune-nude mouse model.

## 2. Materials and Methods

### 2.1. Experimental Animals

Athymic nude mice were purchased from the Charles River (Kinston, NY) and were incorporated into experiments at 6 weeks of age. This study was approved by the Institutional Animal Care and Use Committee of Memorial Sloan-Kettering Cancer Center. The study complied with the principles of Laboratory Animal Care (NIH publication number 85-23, released in 1985) and also conformed to the ARVO Statement for the Use of Animals in Ophthalmic and Vision Research.

### 2.2. Uveal Melanoma Cell Line

Melanoma cell line C918 used in this animal study was isolated from a choroidal melanoma patient with liver metastasis at the University of Iowa. This cell line was provided by Dr. Robert Folberg (University of Illinois, Chicago) [10, 11]. C918 cell line is a highly invasive, metastatic, and aggressive melanoma cell line. Melanoma cells in this cell line are epithelioid cells in morphology with round nuclei and prominent nucleoli [10, 11]. Cells were cultured in RPMI 1640 Medium with 10% fetal bovine serum and 1% penicillin/streptomycin (all from Gibco; Grand Island, NY, USA). Cells were trypsinized and resuspended in the above medium and held on ice until inoculation.

### 2.3. Inoculation of Melanoma Cells and Zeaxanthin Treatment

Mice were randomly divided into three groups, zeaxanthin high dose group (14 eyes) zeaxanthin low dose group (14 eyes), and the control group (not treated with Zeaxanthin, 14 eyes). The methods for inoculation of tumor cells into the posterior segments of the eye have been described previously [12, 13]. Briefly, nude mice were anesthetized by intraperitoneal injection of a ketamine (final concentration; 10 mg/mL) and xylazine (final concentration; 1 mg/mL) mixture (0.01 mL/g mouse weight) and with Alcaine (proparacaine HCL) ocular surface anesthesia. Under a surgical microscope, a 30-gauge sharp needle was used to make two holes through the sclera, one into the intravitreal space to reduce intraocular pressure and one tangentially through the sclera into the subretinal space for injection. Uveal melanoma cells (1 × 10^6^ cells) were injected through the second hole into the choroid and subretinal space using a 1.5 cm, 33-gauge blunt end microinjection needle (7803-05, Hamilton, Reno, NV). After the injection, eyes were covered with ophthalmic bacitracin ointment and buprenorphine was administrated for controlling of pain [12, 13]. Zeaxanthin (supplied by ZeaVision LLC; Chesterfield, MO, USA), solved with DMSO and diluted by PBS, was coinjected with the cellular suspension. The dosages were 114 *μ*g in the low dose group and 570 *μ*g in the high dose group. DMSO at the same levels as zeaxanthin treated group was injected into the eyes in the control group. The mice were kept under sterile conditions in laminar air-flow clean benches at room temperature (25–28°C) and a relative humidity of 55%. Sterile food pellets and water were given. Mice were examined by dissecting microscopy. One week after inoculation of melanoma cells, mice were treated by intravitreous injection of zeaxanthin. Mice were anaesthetized by isoflurane inhalation. Zeaxanthin was solved with DMSO at 50 mM and 57 *μ*g of zeaxanthin was injected into vitreous of mice eyes with 31 G needle in zeaxanthin low group and 114 *μ*g of zeaxanthin in high group. Control groups were injected with 2 *μ*L of DMSO. After 21 days, mice were sacrificed by CO_2_ asphyxiation and the eyes enucleated.

### 2.4. Gross Examination and Measurement of Tumor Mass

Enucleated eyeballs were examined grossly. Extraocular tissue was removed and tumor-bearing eye mass determined. Tumor mass was calculated by the mass of the eye minus the average mass of control uninjected eyes.

### 2.5. Microscopic Examination and Measurement of Tumor Size

The methods for the fixation of the eye have been reported previously [13]. Briefly, the tumor-bearing eyes were fixed overnight at 4°C in 4% paraformaldehyde in PBS (PFA/PBS), incubated in 30% sucrose/PBS overnight at 4°C, embedded in one-part 30% sucrose/PBS and two-part optimal cutting temperature compound (OCT; Miles Laboratories, Elkhart, IN), frozen, and sectioned at 5 to 7 *μ*m [13]. Slides were fixed with Rapid Fixative (Poly Scientific R&D Corp., Bay Shore, NY). Hematoxylin and eosin (HE) staining was carried out using Leica HE Stainer (Leica Biosystems, Buffalo Grove, IL). HE stained sections were examined by a senior ophthalmic pathologist (CI) and a senior uveal melanoma researcher (DNH) to determine the presence and the extent of melanoma. Microscopic photography of eye section was taken using Olympus BX 41 light microscope (Shinjuku, Tokyo, Japan). Tumor size was determined by using of Adobe Photoshop CS6 [14, 15].

## 3. Results

### 3.1. Gross Examination and Tumor Mass

**Figure 1:**
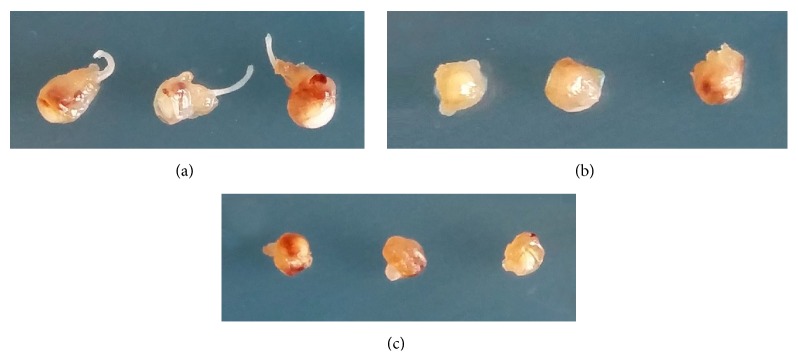
Photographs of enucleated mouse eyes inoculated with human uveal melanoma cells with or without zeaxanthin treatment. Eyes not treated with zeaxanthin (a) show enlargement of the eyeball and with visible extraocular extension of melanoma in some eyes. Eyes treated with zeaxanthin at low dosages (b) or high dosages (c) do not have extraocular extension of melanoma and most of eyeballs are normal in size.

Gross examination revealed that the eyeballs were enlarged in controlled eyes (Figure 1(a)). Half of the eyes had visible extraocular extension of melanoma under stereomicroscope. Most of the zeaxanthin treated eyes were normal in size and were without extraocular extension of melanoma (Figures 1(b) and 1(c)).

Both the eye mass and tumor mass in the eyes of control group were significantly greater than those in eyes of zeaxanthin treated groups (Tables 1 and 2). Furthermore, the eye mass and tumor mass in the eyes of zeaxanthin low group were significantly higher than those in the eyes in zeaxanthin high group (Tables 1 and 2).

### 3.2. Microscopic Examination

**Figure 2:**
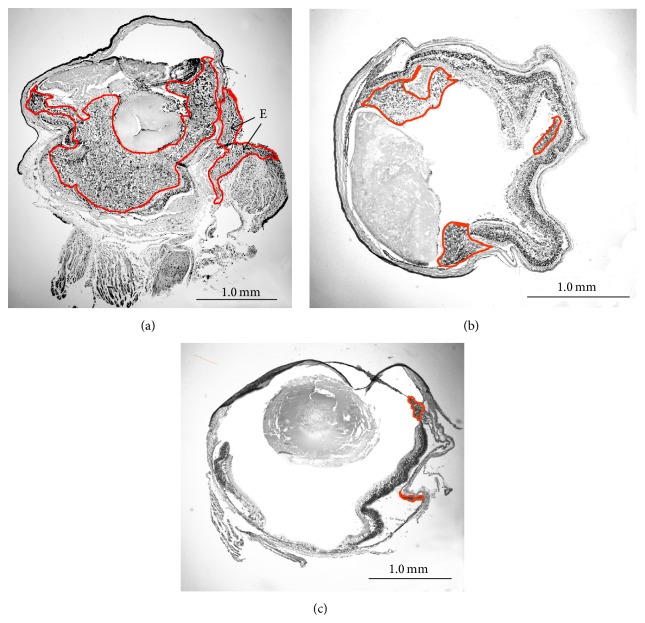
Microscopic photographs of mouse eyes inoculated with human uveal melanoma cells with or without zeaxanthin treatment (original magnification ×4). Eyes were enucleated and stained with hematoxylin-eosin in frozen sections. Tumor was marked by red outlines. In the eye not treated with zeaxanthin (control eye), tumor fills large part of the eyeball (a) with scleral perforation and extraocular extension of melanoma (arrow E). Tumor in eye treated with low dosage of zeaxanthin (b) is smaller than that of the control eye. Tumors in eye treated with high dosage of zeaxanthin (c) are much smaller than that of eye treated with low dosage of zeaxanthin and control eye.

**Figure 3:**
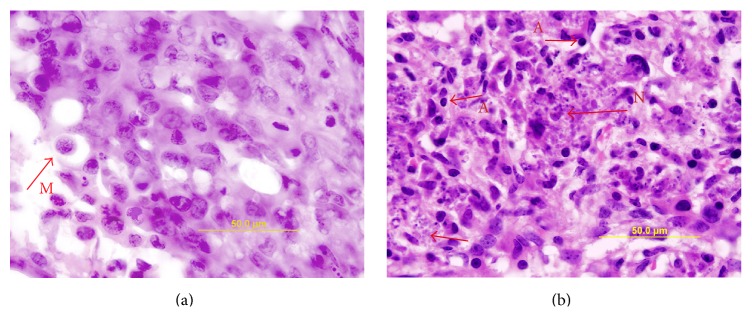
Microscopic photographs of mouse eyes inoculated with human uveal melanoma cells with or without zeaxanthin treatment (original magnification ×100). Eyes were stained with hematoxylin-eosin in frozen section and observed under oil lens. Tumor cells in the eye not treated with zeaxanthin are mostly epithelioid in morphology with few spindle cells. Large nuclei and prominent nucleoli were observed in the tumor cells (a). Mitoses are common (arrow M). In eyes treated with high dosage of zeaxanthin, necrotic (arrow N) or apoptotic tumor cells (arrow A) can be observed. No mitotic figures are present (b).

Melanoma grew rapidly in the control eyes (melanoma cells inoculated without zeaxanthin treatment). Microscopic examination confirmed the presence of large melanoma xenografts filling the eyes of most control mice (Figure 2(a)). Half of the eyeballs had definitely scleral perforation and extraocular extension of melanoma cells (Figure 2(a)). Tumor cells in the mouse eye were mostly epithelioid in morphology with few spindle cells. Large nuclei and prominent nucleoli were observed in the tumor cells. Mitoses were common (Figure 3(a)).

Tumors in zeaxanthin low group were smaller than those of the control group (Figure 2(b)). The tumor cells were epithelioid or spindle in morphology with large nuclei and prominent nucleoli. Mitoses were observed occasionally. Necrotic or apoptotic tumor cells were present in part of the eyes. Scleral invasion and extraocular extension of melanoma have not been found in this group.

Tumors in zeaxanthin high group were much smaller than those of the zeaxanthin low group and control group (Figure 2(c)). Patches of definite melanoma cells could be found only in approximately two-thirds of eyes. No miotic figures were present. Necrotic or apoptotic tumor cells could be found in most eyes (Figure 3(b)) and no scleral invasion and extraocular extension of melanoma were present in this group.

### 3.3. Tumor Size

Tumor size was 1.69 ± 0.95 mm^2^ (mean ± standard deviation), 0.74 ± 0.55 mm^2^, and 0.13 ± 0.13 mm^2^ in the control group, zeaxanthin low group, and zeaxanthin high group, respectively. The difference of tumor size between these three groups was statistically significant (*p* < 0.001). The tumor sizes in the control group were significantly greater than those in both zeaxanthin high and low groups (both *p* < 0.0001), whereas the tumor size in eyes treated with high dosage of zeaxanthin was significantly smaller than that in mice treated with low dose of zeaxanthin (*p* < 0.05). Using the tumor size of control group as 100%, the tumor sizes in zeaxanthin low group and zeaxanthin high group were 43.9% and 7.7%, respectively (Table 3).

## 4. Discussion

Our previous study demonstrated that zeaxanthin significantly inhibits the growth and induces apoptosis of human uveal melanoma cells* in vitro* [10]. However, the results of* in vitro* study may or may not accurately predicate the results obtained from* in vivo* study. For example, it has been reported that interleukin-1 (IL-1) may play a role in promoting uveal melanoma progression. However, inhibiting IL-1 with IL-1ra (an antagonist of IL-1) slows tumor growth only* in vivo* but not* in vitro* [16].* In vitro* studies test only the direct effects of a medication on the tumor cells.* In vivo* studies test the effects of the medication on the production of various bioactive factors produced by tumor cells or neighbor cells, which in turn may affect the growth and invasion of tumor* in vivo* (paracrine effect), in addition to its direct effects. For example, angiogenesis plays an important role in the growth and progress of uveal melanoma. VEGF is a potent stimulator for angiogenesis. The results of several previous studies suggested that zeaxanthin inhibits the production of VEGF by various ocular cells or inflammatory cells [17–21]. This may reduce the angiogenesis and results in the inhibition of the growth of uveal melanoma* in vivo*.* In vivo* studies are an important component of preclinical evaluation of any therapeutic approach to the clinical management of patients with uveal melanoma. For this reason, we designed and carried out the current study for testing the effects of zeaxanthin on the growth and invasion of human uveal melanoma* in vivo* using a nude mouse model.

Numerous animal models have been developed and used in the* in vivo* study of uveal melanoma. The melanoma cells used could be experimental animal melanoma cells (Greene hamster or B16 mouse melanoma cell lines) [22] or human melanoma cells [22–44]. Use of human melanoma cells has the advantage of avoiding the species difference and may more accurately reflect the biological behavior of uveal melanoma in the patients. Tumor cells are antigenic and can induce immune rejection of inoculated tumor graft, especially in transplantation of human tumor cells into experiment animals (xenografts). It has been reported that immune privilege is present in the anterior chamber of the eye, permitting melanoma xenografts to survive if inoculated into the rabbit's anterior chamber [22]. However, since this privilege is incomplete, therefore, in order to grow human melanoma cells in an experiment animal model, it is necessary to use animals incapable of mounting immune rejection to xenograft tumor cells [23–44]. This can be achieved by using immune inhibitory drugs [41–44] or inoculate tumor cells into immune incompetent nude mice [23–40]. The nude mouse is a hairless mutant born without a thymus, which causes a severe defect in cellular immunity, that is, in the transformation process of T lymphocyte precursors to functional T cells. Nude mice have the ability to accept human melanoma cells while preserving many human uveal melanoma characteristics [32]. Therefore, uveal melanoma xenografts in nude mice are a widely used model for studying melanoma growth and response to therapeutic interventions [23–40].

Melanoma cells can be inoculated intraocularly (orthotopic model) [22–31] or subcutaneously (heterotopic model) [32–38]. Tumors transplanted to heterotopic sites may not display biological behavior consistent with the original tumor. The difference of biological behaviors between orthotopic and heterotopic transplantations may be related to the influence of local organ-specific factors. Therefore, the importance of orthotopic, rather than heterotopic, transplantation cannot be overemphasized [24].

The site for intraocular inoculation of melanoma cells could be the anterior part (anterior chamber) [23–25, 29–31, 44] or the posterior part of the eye (vitreous, choroid, subretinal, or suprachoroidal space) [26–28, 30, 45, 46]. Uveal melanoma may arise clinically in the iris (anterior part) or in the ciliary body/choroid (posterior part). Most iris melanomas are relatively benign and only account for approximately 5% of uveal melanoma, which is different from the relatively poor prognosis for patients with melanoma of the ciliary body or choroid [45]. Therefore, we selected the inoculation of melanoma cells into the posterior segment. We ideally inoculated the cells into the choroid; however, in such tiny eyes it is virtually impossible to direct the cells only into the choroid; some cells may enter the suprachoroidal space, subretinal space, or the vitreous [45].

Human uveal melanoma cells used in the present study are the C918 melanoma cell line, which was isolated from a choroidal melanoma patient with liver metastasis. Melanoma cells in this cell line are epithelioid in morphology with round nuclei and prominent nucleoli [10, 11]. The morphologic phenotype of a uveal melanoma provides an important indication of malignancy. The Challender classification scheme categorizes uveal melanoma cellular components as either spindle A, spindle B, or epithelioid. A uveal melanoma predominance of epithelioid components carries significantly greater malignant potential and a shorter patient survival time than melanomas comprised largely spindle cellular elements [47]. C918 cell line is a highly invasive, metastatic, and aggressive melanoma cell line* in vitro* and has been used previously in several animal studies of uveal melanoma [28, 32, 33]. In the present study, melanoma developed rapidly and had potent invasive capacity in mice inoculated with C918 cells and these cells also showed the epithelioid morphology, indicating that this melanoma model reflects the biological behavior of uveal melanoma* in vitro* and in patients with uveal melanoma quite well.

In the present study, melanoma developed in mice without the treatment of zeaxanthin. Melanoma grew rapidly to occupy a large part of the eye and extraocular extension occurred in one-half of the eyes. In zeaxanthin treated groups, zeaxanthin was injected to the posterior part of the eye twice with a total dosage of 171 *μ*g (zeaxanthin low group) or 684 *μ*g (zeaxanthin high group). Zeaxanthin treatment significantly inhibited the growth and invasion of melanoma in nude mice eyes, especially in zeaxanthin high group. Gross examination and histopathological examination found that the tumor mass and size in zeaxanthin treated eyes were significantly less than those in the controls and the extraocular extension only occurred in eyes without the treatment of zeaxanthin. Numerous necrotic or apoptotic tumor cells could be found in eyes treated with zeaxanthin. Quantitative histopathological study demonstrated that the tumor size was reduced by 56% in zeaxanthin low group and 92% in zeaxanthin high group as compared to the control group. All of these results are consistent with those in our previous* in vitro* study which demonstrated the growth inhibition and apoptosis induced effects of zeaxanthin on cultured human uveal melanoma cells.

The dosages used in the animal study have been calculated and compared to the dosages used in the* in vitro* study. In the low dosage group of the animal study, 1 × 10^6^ cells were injected into the eye, and the dosage of zeaxanthin used was 114 *μ*g (first injection) added to 57 *μ*g (second injection); therefore, the total dosage used was 171 *μ*g of zeaxanthin per 1 × 10^6^ cells. The tumor mass in eyes treated with this dosage was 58% of the control (reduced by 42%). In the high dosage group, the total dosages used were 570 *μ*g (first injection) added to 114 *μ*g (second injection); therefore, the total dosage used was 684 *μ*g of zeaxanthin per 1 × 10^6^ cells. The tumor mass in eyes treated with this dosage was 32% of the control (reduced by 68%). In the* in vitro *study, the ID50 dosage of zeaxanthin in C918 cells was 28.7 *μ*M [10]. In that study, 5 × 10^3^ cells were tested in 96 wells with 200 *μ*L of culture medium containing 28.7 *μ*M zeaxanthin, which equals 3.26 *μ*g of zeaxanthin [10]. Therefore, the dosage of zeaxanthin that can reduce the cell viability to 50% of the control was 3.26 *μ*g zeaxanthin/5 × 10^3^ cells, which equals 652 *μ*g of zeaxanthin per 1 × 10^6^ cells, slightly lower than that used in the high dosage group but greater than in the low dosage group in the animal study. Therefore, the dosages in the animal study are consistent with the dosages used in the* in vitro* study.

It has been reported that zeaxanthin can inhibit the growth and/or induced apoptosis in lymphoma, breast cancer, and neuroblastoma cells* in vitro* [48, 49]. Zeaxanthin had moderate effects in reversing multidrug resistance in mouse lymphoma and human breast cancer cells [48, 50]. Zeaxanthin inhibited the invasion of rat ascites hepatoma cells* in vitro* [51]. Baudelet et al. reported that the extracts of the Glaucophyte* Cyanophora paradoxa *could inhibit the growth of cutaneous melanoma, mammary carcinoma, and lung adenocarcinoma cells* in vitro*. Further analysis indicated that zeaxanthin was one of the three main pigments or derivatives responsible for the cytotoxicity of* Cyanophora paradoxa *fractions in cancer cells [52]. For the experimental animal study, Firdous et al. reported that oral administration of meso-zeaxanthin, another xanthophyll carotenoid, could significantly increase tumor latency period in 3-methylcholanthrene-induced sarcoma in mice. Survival of tumor-bearing mice was significantly increased by meso-zeaxanthin treatment [53]. All of these results are consistent with the results from the present study.

In conclusion, we have demonstrated in the present* in vivo* study that intraocular administration of zeaxanthin significantly inhibits the growth and invasion of human uveal melanoma in nude mice. The results of the present study may be useful for the development of a novel therapeutic approach to the management of uveal melanoma, especially for the combination of zeaxanthin with other aggressive uveal melanoma treatments.

## Figures and Tables

**Table 1:** Comparison of tumor-bearing eye mass in different groups.

Eye mass	Control	ZL	ZH
Mean (mg, mean ± SD)	21.3 ± 3.5	16.1 ± 3.4	12.4 ± 3.2
Percentage	100%	76%	58%

Control: mice not treated with zeaxanthin; ZL: zeaxanthin low group; ZH: zeaxanthin high group; one-way ANOVA, *p* < 0.001; ZL: control, *p* < 0.001; ZH: control, *p* < 0.001; ZL: ZH, *p* < 0.05.

**Table 2:** Comparison of tumor mass in different groups.

Eye mass	Control	ZL	ZH
Mean (mg, mean ± SD)	12.3 ± 3.5	7.1 ± 3.4	3.4 ± 3.2
Percentage	100%	58%	32%

Control: mice not treated with zeaxanthin; ZL: zeaxanthin low group; ZH: zeaxanthin high group; one-way ANOVA, *p* < 0.001; ZL: control, *p* < 0.001; ZH: control, *p* < 0.001; ZL: ZH, *p* < 0.05.

**Table 3:** Comparison of tumor size in different groups.

Tumor size	Control	ZL	ZH
Mean (mm^2^, mean ± SD)	1.70 ± 0.95	0.74 ± 0.55	0.13 ± 0.12
Percentage	100%	44%	7.7%

Control: mice not treated with zeaxanthin; ZL: zeaxanthin low group; ZH: zeaxanthin high group; one-way ANOVA, *p* < 0.001; ZL: control, *p* < 0.001; ZH: control, *p* < 0.001; ZL: ZH, *p* < 0.05.
